# Targeting the 5-HT7 receptor to block influenza virus entry: mechanistic insights and therapeutic potential of SB269970 HCl

**DOI:** 10.3389/fmicb.2026.1718860

**Published:** 2026-03-25

**Authors:** Yarou Gao, Xingjian Zhu, Jiaxin Ke, Yue Su, Xiaoqin Lian, Lefang Jiang, Mingxin Zhang, Yang Yu, Qun Peng, Xulin Chen

**Affiliations:** Institute of Medical Microbiology, Department of Immunology and Microbiology, College of Life Science and Technology, Jinan University, Guangzhou, China

**Keywords:** 5-HT7 receptor, antiviral activity, *in vitro*, *in vivo*, influenza virus, SB269970 HCl, viral endocytosis

## Abstract

Influenza viruses persist as a significant global health challenge, with the emergence of drug-resistant strains limiting the effectiveness of current antiviral treatments. Inhibiting the early stages of viral replication is known to reduce virus-induced cytopathic effects; however, few therapeutics target these stages or the host factors supporting them, creating a critical gap in anti-influenza strategies development. This study aimed to address this gap by evaluating the antiviral potential of SB269970 HCl, a 5-HT7 receptor antagonist, against influenza viruses. We tested the efficacy of the compound using *in vitro* models (with multiple influenza strains) and an *in vivo* murine model of lethal influenza infection. *In vitro*, SB269970 HCl effectively suppressed viral replication, and *in vivo*, it significantly improved survival rates and reduced the viral load in infected mice. Mechanistic experiments revealed that SB269970 HCl exerts its antiviral activity by blocking endocytosis of the influenza virus, a key step in the viral life cycle. Genetic knockdown of the 5-HT7 receptor inhibited viral entry, demonstrating the pivotal role of this receptor in facilitating viral internalization. These findings advance anti-influenza research by identifying the 5-HT7 receptor and its associated pathways as novel druggable targets. Unlike traditional antivirals, which are prone to resistance, SB269970 HCl targets host factors, offering a broad-spectrum strategy effective against diverse influenza strains, and establishing it as a promising candidate for further antiviral development.

## 1 Introduction

Influenza viruses present a significant global health threat, causing seasonal outbreaks and occasional pandemics. Seasonal influenza epidemics lead to hundreds of thousands of deaths and high rates of illness worldwide (Dhanasekaran et al., 2022). In contrast, pandemics are associated with even higher rates of illness and death, along with considerable economic impact (Tscherne and Garcia-Sastre, 2011; Kumari et al., 2023). These viruses are capable of adapting to different hosts and undergoing genetic reassortment, which contributes to the ongoing emergence of new strains with unpredictable pathogenicity, transmissibility, and potential for pandemics (Tscherne and Garcia-Sastre, 2011). Although vaccines and antiviral medications are available, the rapid mutation rates of influenza viruses allow them to evade immune defenses and develop resistance to these treatments. This highlights the urgent need for novel therapeutic approaches (Hayden et al., 2018; Petrova and Russell, 2018).

Current antiviral treatments primarily focus on targeting key viral proteins, such as neuraminidase (NA) and M2 ion channels, which are vital for viral replication and release (Jefferson et al., 2014). Drugs such as oseltamivir (Tamiflu) and zanamivir (Relenza) inhibit neuraminidase activity and are commonly used to treat influenza infections. However, the emergence of drug-resistant strains, particularly those with mutations in the NA or M2 genes, has significantly diminished the effectiveness of these treatments (Hayden et al., 2003; Deyde et al., 2007). For instance, widespread resistance to adamantanes, a category of M2 inhibitors, has rendered these medications largely ineffective against current strains of influenza A (Bright et al., 2005). Furthermore, existing antiviral drugs often fail to target early stages of viral replication, such as viral endocytosis, which are crucial for establishing an infection and initiating cytopathic effects in host cells (Schelhaas, 2010; Barrow et al., 2013). These processes include clathrin-mediated endocytosis, caveolar endocytosis, macropinocytosis, and other pathways (Sieczkarski and Whittaker, 2002).

Focusing on the early phases of the viral life cycle offers a promising approach to overcome the challenges associated with existing therapeutic methods. Influenza viruses rely on host cellular processes for entry and replication, making host factors suitable targets for therapeutic interventions (Karlas et al., 2010). By blocking host factors that facilitate viral entry, researchers can create broad-spectrum antiviral drugs that are less likely to lead to resistance (Ji and Li, 2020). Recent studies have highlighted the potential of targeting host receptors and signaling pathways involved in viral uptake, thus laying the groundwork for novel strategies to combat influenza infections (Ludwig, 2011; Kuck et al., 2014; Du et al., 2015; Droebner et al., 2017).

Serotonin receptors (5-HTRs), classified as G protein-coupled receptors (GPCRs), have recently emerged as significant candidates for investigation in various research fields. Historically, these receptors have primarily been studied for their roles in central nervous system functions, including mood regulation and circadian rhythms. However, contemporary research underscores the potential involvement of 5-HTRs in viral entry mechanisms (Cheng et al., 2015; Mainou et al., 2015; Riva et al., 2018; Bouma et al., 2020; Mayberry et al., 2021). Notably, the 5-HT7R subtype has been recognized as a viable drug target for inhibiting hepatitis B infection by activating the AC/PKA/ERK signaling pathways, a process facilitated by modulation of DDX3 expression (Kang et al., 2019). Given the integral role of serotonin receptors in viral replication, investigating their potential as targets for inhibiting influenza virus replication may represent a promising strategy for antiviral interventions.

SB269970 HCl (SB), a selective antagonist of the 5-HT7 receptor, has been extensively characterized for its ability to inhibit receptor signaling and its subsequent effects in both neuronal and non-neuronal systems (Thomas and Hagan, 2004; Lieb et al., 2005; Monti and Jantos, 2014). Nonetheless, its potential role in inhibiting viral replication remains largely unknown. Our recent research found that SB has antiviral effects against the influenza virus in U937 cells (Gao et al., 2025). Nonetheless, the potential of SB Cl as an antiviral agent and its mode of action against influenza viruses remain to be fully explored.

In this study, we sought to evaluate the antiviral efficacy of SB against influenza *in vitro and in* murine models. Our objective was to investigate the mechanism by which SB inhibits influenza virus replication and determine whether the 5-HT7 receptor (5-HT7R) is a target of this inhibition. Our findings demonstrate that SB significantly reduces viral replication across multiple strains by interfering with the virus’s internalization process. This observed inhibition correlates with significant survival benefits and reduced viral load in mice subjected to lethal infections, underscoring its therapeutic potential. Furthermore, genetic knockdown of 5-HT7R exhibited a marked antiviral effect. Investigations into the mechanism of action revealed that treatment with SB and knockdown of 5-HT7R impeded influenza virus endocytosis. These results demonstrate the potential of SB as an entry inhibitor for influenza virus infections. They also improve our understanding of the role of 5-HT7R in cellular physiology, positioning it as a novel host target for tackling the challenges posed by influenza’s resistance to direct-acting antiviral agents.

## 2 Results

### 2.1 SB inhibits the replication of multiple influenza virus strains *in vitro*

**Figure 1:**
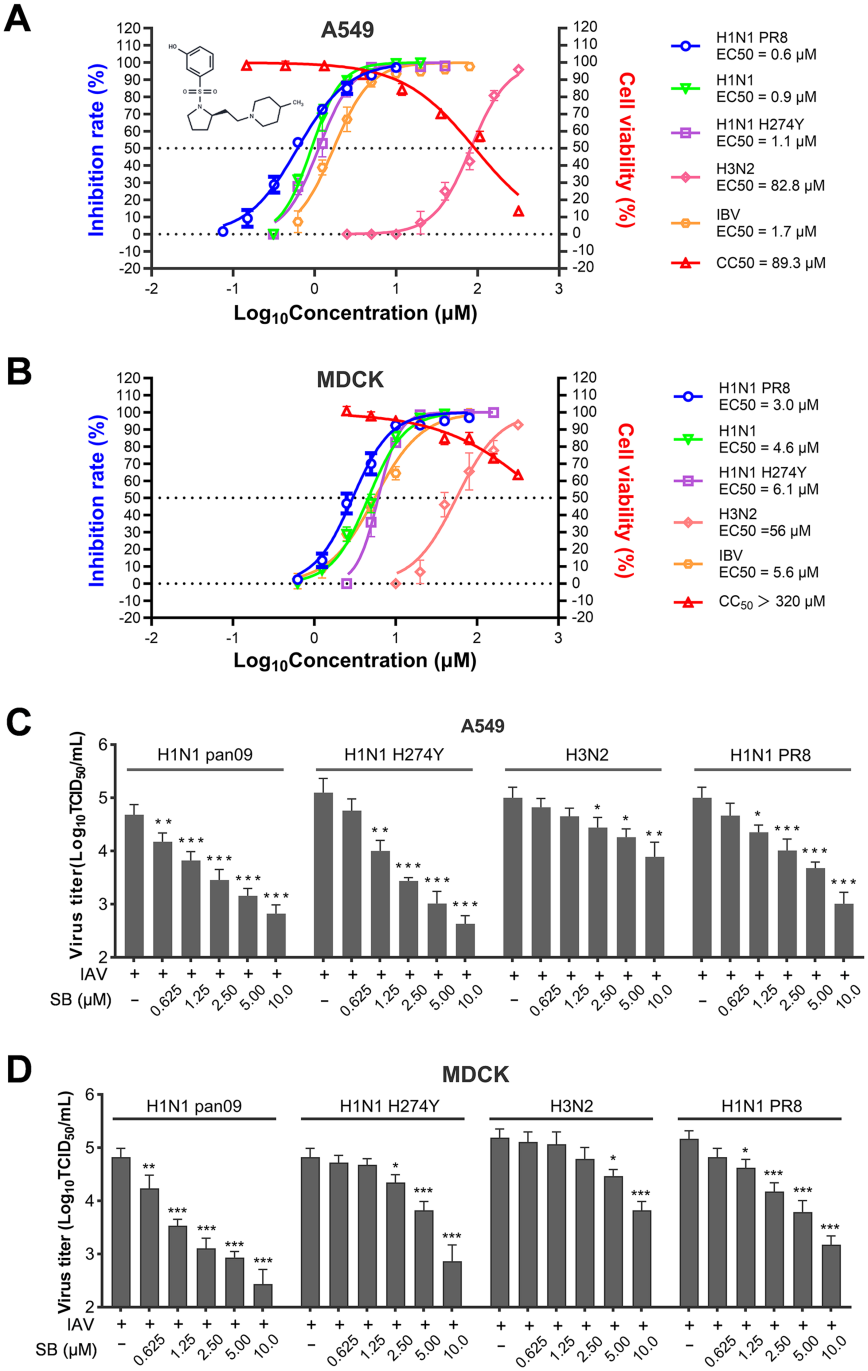
The cytotoxicity and broad-spectrum antiviral effects of SB against various influenza virus strains in A549 and MDCK cells. **(A,B)** The chemical structures of SB are shown in **A**. A549 and MDCK cells, cultured in 96-well plates, were infected with different influenza virus strains (H1N1 pan2009, H1N1 OST-R, H3N2, PR8, and IBV) either in the absence or presence of varying concentrations of SB at 37 °C for 1 h. Subsequently, the cells were treated with fresh medium containing different concentrations of SB. Following 24 h of incubation at 37 °C, the supernatants were collected, and the antiviral effects of SB were evaluated using an NA activity assay. Cell viability was assessed using the CellTiter-Glo kit after 48 h of exposure to SB at the specified concentrations. **(C,D)** A549 and MDCK cells were cultured in 24-well plates and infected with multiple IAV strains (H1N1 pan2009, H1N1 OST-R, H3N2, and PR8) in the absence or presence of different concentrations of SB, followed by a 1-h incubation at 37 °C. The cells were then treated with fresh medium containing various concentrations of SB. Supernatants and cells were collected after 24 h, and virus titers were determined using the TCID_50_ method on MDCK cells. The data presented represent results from three independent experiments (mean ± SD). Statistical significance was analyzed using one-way analysis of variance (ANOVA). Statistically significant differences are indicated as ^*^p < 0.05, ^**^p < 0.01, and ^***^p < 0.001.

In prior investigations, we identified SB as possessing an inhibitory effect on the replication of the PR8 strain in U937 cells (Gao et al., 2025). To further assess the *in vitro* antiviral efficacy, we examined antiviral activities of SB on both A549 and MDCK cell lines against a range of influenza virus strains. As shown in Figures 1A,B, our findings indicate that SB effectively inhibited the replication of three type A influenza virus strains, including the H1N1 PR8 strain, an oseltamivir-resistant H1N1 PR8 strain, a clinically circulating H1N1 seasonal flu strain, and a type B influenza virus strain in both cell lines. The calculated EC_50_ values, determined based on the virion-associated neuraminidase activity of the supernatant, varied from 0.6 to 1.7 μM in A549 cells and from 3.0 to 6.1 μM in MDCK cells.

Nevertheless, SB exhibited significantly lower efficacy against the type A influenza virus H3N2 strain, with EC_50_ values of 82.8 μM on A549 cells and 56 μM on MDCK cells. Additionally, SB demonstrated mild cytotoxicity toward both cell lines, with a CC_50_ value of 89.3 μM in A549 cells and a figure slightly exceeding 320 μM in MDCK cells. Consequently, these results confirm that SB possesses considerable antiviral activity against multiple influenza A and B virus strains, yielding a selectivity index ranging from 52 to 148. However, it is noteworthy that SB does not inhibit the replication of the H3N2 virus strain in A549 cells, and its effectiveness against the H3N2 virus in MDCK cells is markedly low, with a selectivity index of 5.7.

The antiviral efficacy of SB was further validated by measuring viral titers in the supernatants of cell cultures treated with serial concentrations of the compound. As illustrated in Figures 1C,D, treatment with SB resulted in a concentration-dependent inhibition of influenza virion production across all tested strains. The calculated EC_50_ values, determined from the viral titers in the supernatant, were 0.6, 0.8, 33, and 1.7 μM in A549 cells, and 2.6, 2.8, 30, and 3.7 μM in MDCK cells. Collectively, these findings indicate that SB effectively inhibits the replication of type A influenza viruses, including a strain resistant to oseltamivir. However, it exhibits significantly weaker inhibition against the type A H3N2 influenza virus.

### 2.2 SB inhibits viral replication and protects mice from the lethal influenza virus infection

**Figure 2:**
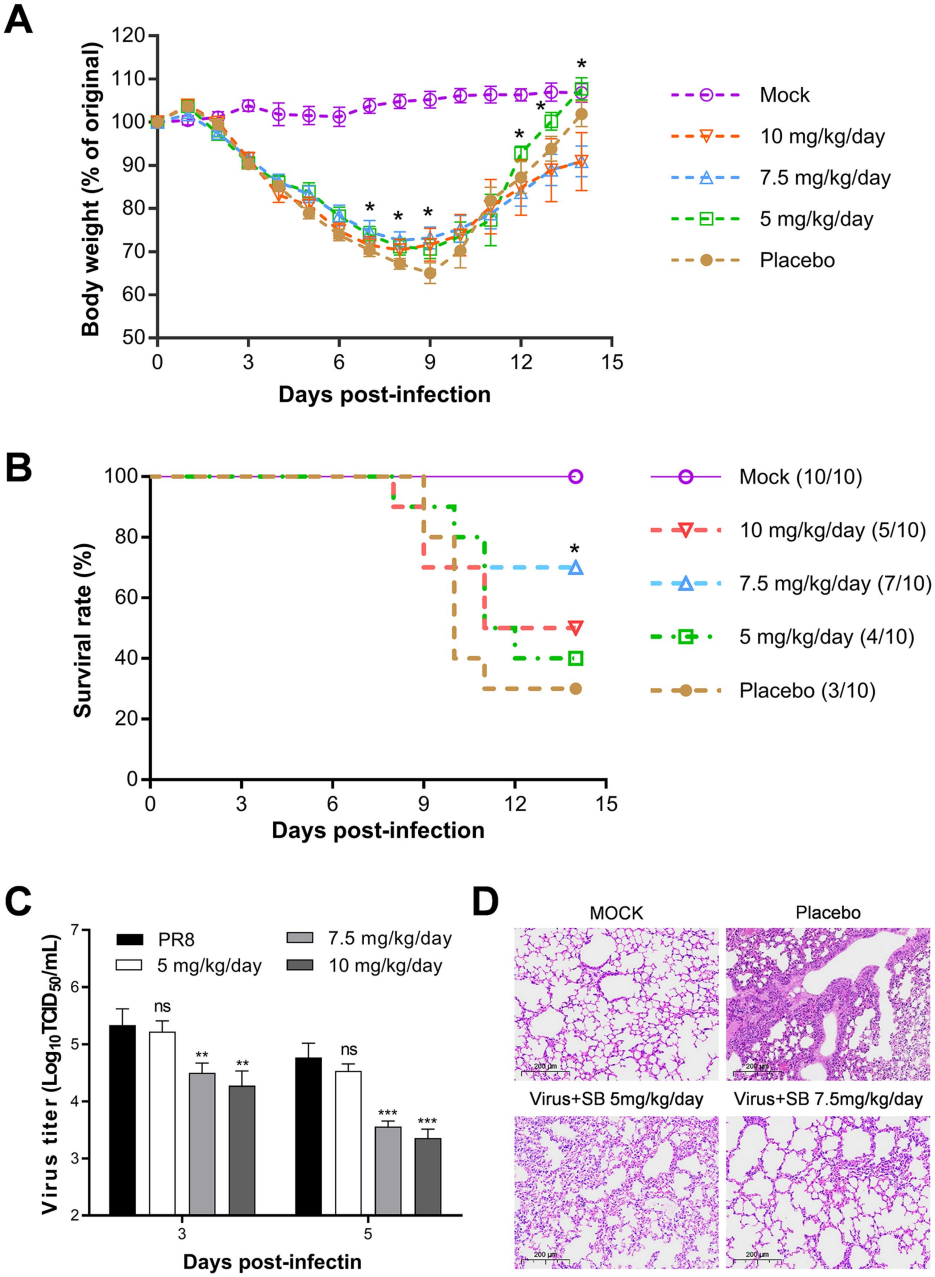
Evaluation of the *in vivo* antiviral efficacy of SB against lethal influenza infection. BALB/c mice (*n* = 10) received varying doses of SB (5, 7.5, or 10 mg/kg) or 2.5% DMSO before being infected with PR8 (5 × LD_50_ in 30 μL of PBS) via intranasal inoculation. Post-infection, the mice were treated intraperitoneally with SB or 2.5% DMSO twice daily for 3 days. Over 14 days, the following parameters were monitored and recorded: **(A)** body weight (*n* = 10) and **(B)** survival rate (*n* = 10). Additionally, **(C)** viral titers in the lungs (*n* = 3) were assessed using TCID_50_ on days 3 and 5 post-infection. **(D)** Microscopy images of lung tissue sections, stained with hematoxylin and eosin, were obtained on day 5 post-infection, with a scale bar of 200 μm. Statistically significant differences are indicated as ^*^p < 0.05, ^**^p < 0.01, and ^***^p < 0.001.

We evaluated the therapeutic efficacy of SB in a mouse model of influenza. In this study, 6–8-week-old BALB/c mice were infected with a dose of 5 × LD_50_ of the PR8 virus, resulting in a 70% mortality rate among infected subjects. The mice receiving the viral infection were administered SB intraperitoneally at doses of 5, 7.5, and 10 mg/kg/day, or a control solution containing 0.9% sodium chloride and 2.5% DMSO, administered twice daily over the course of 3 days. The initial administration occurred 30 min before the viral infection. As indicated in Figure 2A, mice treated with SB displayed a marginally reduced rate of body weight loss starting from day 3 post-infection (dpi) compared to the placebo group. In the course of our investigation, we established a body weight loss endpoint of 35%. None of the subjects reached the euthanasia threshold of 35%; consequently, all mice, except those that succumbed within 14 days, were observed for the entire duration of 14 days. All surviving mice began to regain body weight from day 9 dpi. Notably, the group treated with 5 mg/kg/day of SB exhibited a more rapid recovery of body weight than the other treated groups. In terms of survival rates, 70% of mice in the placebo cohort succumbed to infection by day 14 post-infection, with a median survival time (MST) of 10 days and a 95% confidence interval (95% CI). Conversely, the survival rates among the 5 mg/kg/day, 7.5 mg/kg/day, and 10 mg/kg/day, SB-treated groups showed improvement, with survival percentages of 40, 70, and 50%, respectively. The MST & 95% CI for these groups were 9, >11, and 9 days, respectively (refer to Figure 2B). However, the differences between the treatment groups and the placebo are not statistically significant. The administration of 10 mg/kg/day of SB was found to be less effective than that of 7.5 mg/kg/day, indicating potential toxicity at the higher dosage. Nevertheless, these findings suggest that treatment with SB confers notable, albeit mild, protection to mice against lethal infection with the influenza virus.

To assess the antiviral efficacy of SB *in vivo*, viral titers in the lung tissue homogenates were quantified utilizing the TCID_50_ assay. As shown in Figure 2C, SB significantly reduced viral titers in a dose-dependent manner at doses exceeding 7.5 mg/kg/day. Specifically, treatment with 10 mg/kg/day of SB resulted in reduction in viral titers of 1.05 and 1.41 on days 3 and 5 post-inoculation, respectively. These findings indicate that SB effectively inhibits influenza virus replication in the lungs of mouse models. Moreover, H&E staining was performed according to the protocols outlined in the Materials and Methods section. The results, depicted in Figure 2D, demonstrated a significant reduction in pulmonary parenchymal and interstitial inflammatory infiltrates in the SB = treated groups compared to the placebo group. Notably, mice treated with 7.5 mg/kg/day of SB exhibited substantial improvements compared with those receiving 5 mg/kg/day of the same treatment.

In summary, our mouse model studies demonstrated that SB has dose-dependent antiviral effects against lethal influenza infection *in vivo*, resulting in reduced lung injuries and protection in mice against lethal influenza virus infection.

### 2.3 SB inhibits the early stage of influenza virus replication

**Figure 3:**
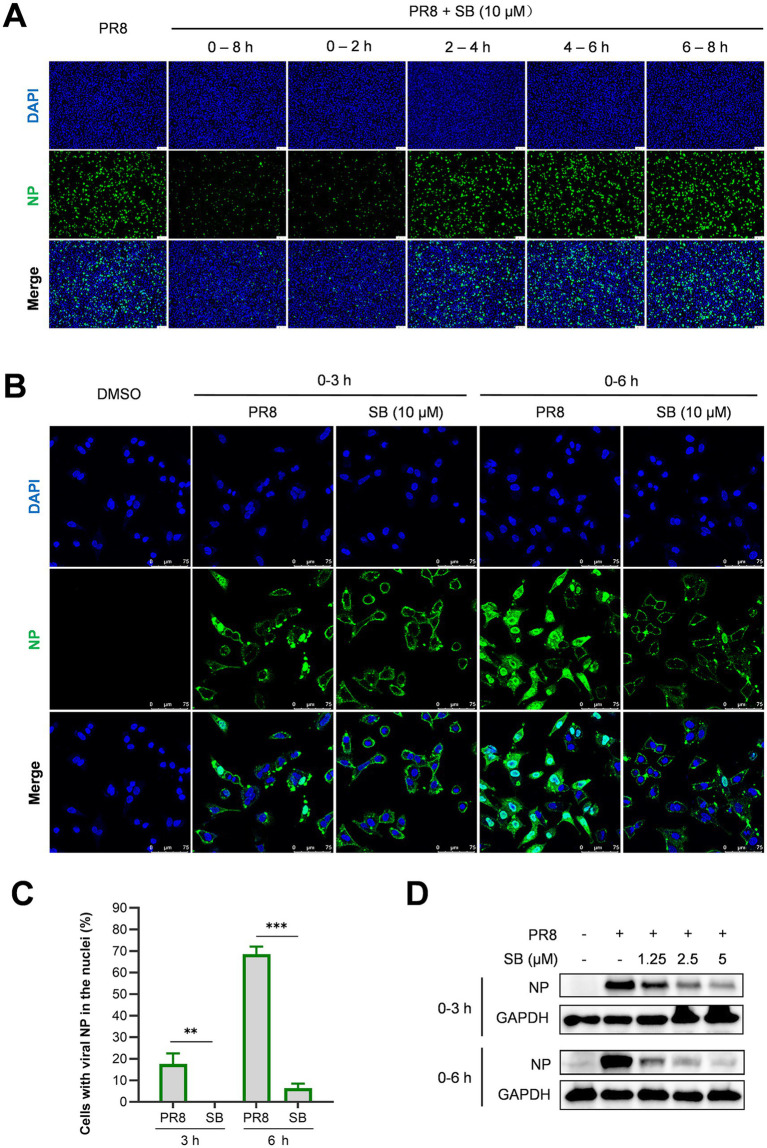
Effect of SB on the stages of the IAV life cycle. **(A)** The specific stage of the viral life cycle affected by SB was evaluated using a time-of-addition assay. A549 cells cultured in multiple wells were infected with 0.5 MOI of PR8 and then either treated with 10 μM SB for the designated duration or left untreated. At 8 hpi, NP expression was analyzed using IFA. The NP was stained green, and the nucleus was visualized with DAPI, which stains the nuclei blue. Scale bar: 100 μm. **(B–D)** Assessment of viral NP localization and expression via IFA and Western blot analysis in A549 cells. Multiple wells of these cells were infected with PR8 at 5 MOI, with or without 10 μM SB. NP localization **(B)**, quantitative analysis from B **(C)**, and NP expression levels **(D)** were evaluated at 3 and 6 hpi using IFA **(B,C)** and Western blotting **(D)**. Scale bar: 75 μm. The proportion of virus-infected cells showing NP localization within the nucleus in **B** was analyzed in over 100 cells. The results presented are representative of three independent experiments (mean ± SD). Statistical significance was determined using one-way analysis of variance (ANOVA). Statistically significant differences are indicated as ^*^p < 0.05, ^**^p < 0.01, and ^***^p < 0.001 in **C**.

To clarify the mechanism by which SB inhibits the influenza virus replication cycle, a time-of-addition assay was conducted to determine which stage of viral replication is affected. The average duration of a complete cycle of influenza virus replication is approximately 8 h, divided into four intervals of 2 h each. Influenza virus-infected cells were treated with SB during each of these four intervals. Cells were also treated throughout their entire lifecycle to serve as a treatment control, while untreated cells acted as a control for viral replication. Levels of NP, assessed via indirect immunofluorescence assay (IFA), represented the extent of viral replication. As shown in Figure 3A, SB treatment during the initial 0–2-h interval completely inhibited viral replication, comparable to the effect observed during the whole 0–8-h treatment period. Conversely, SB treatment within the 2–4-h interval resulted in approximately 20% inhibition of viral replication based on NP levels. However, no inhibition of viral replication was observed following SB treatment at the 4–6 and 6–8 h intervals. This finding suggests that SB exerts its antiviral effects predominantly during the early stages of the influenza virus replication cycle.

To further validate the inhibitory effect of SB on influenza viral replication during the early stages, we assessed the localization and expression of viral NP using IFA at 3 and 6 h post-viral infection (hpi). As shown in Figures 3B,C, which include a quantitative analysis of the results in Figure 3B, at 3 hpi, viral ribonucleoproteins (vRNPs) were detected in the nuclei of 17.6% of virally infected cells in the untreated group. This observation suggests that vRNPs were effectively imported into the nuclei, as indicated by higher fluorescence intensity in newly synthesized viral NP in infected cells. In contrast, SB-treated cells exhibited a complete inhibition of vRNP nuclear import, as evidenced by the absence of NP within the nuclei of infected cells. Similar findings were observed at 6 hpi, with vRNPs were found in the nuclei of 68.5% of the virally infected cells in the untreated group, compared to only 6.4% in the SB-treated group. Given that nuclear import of vRNP is a critical initial step for viral gene expression, we subsequently examined the expression of viral NP through Western blot analysis. NP expression exhibited a dose-dependent reduction following SB treatment (Figure 3D). These findings further reinforce the notion that SB exerts its antiviral effects during the initial phases of the influenza virus replication cycle. Consequently, the nuclear import of viral vRNPs and subsequent stages of viral replication are impacted.

### 2.4 SB inhibits the internalization of influenza virus replication

**Figure 4:**
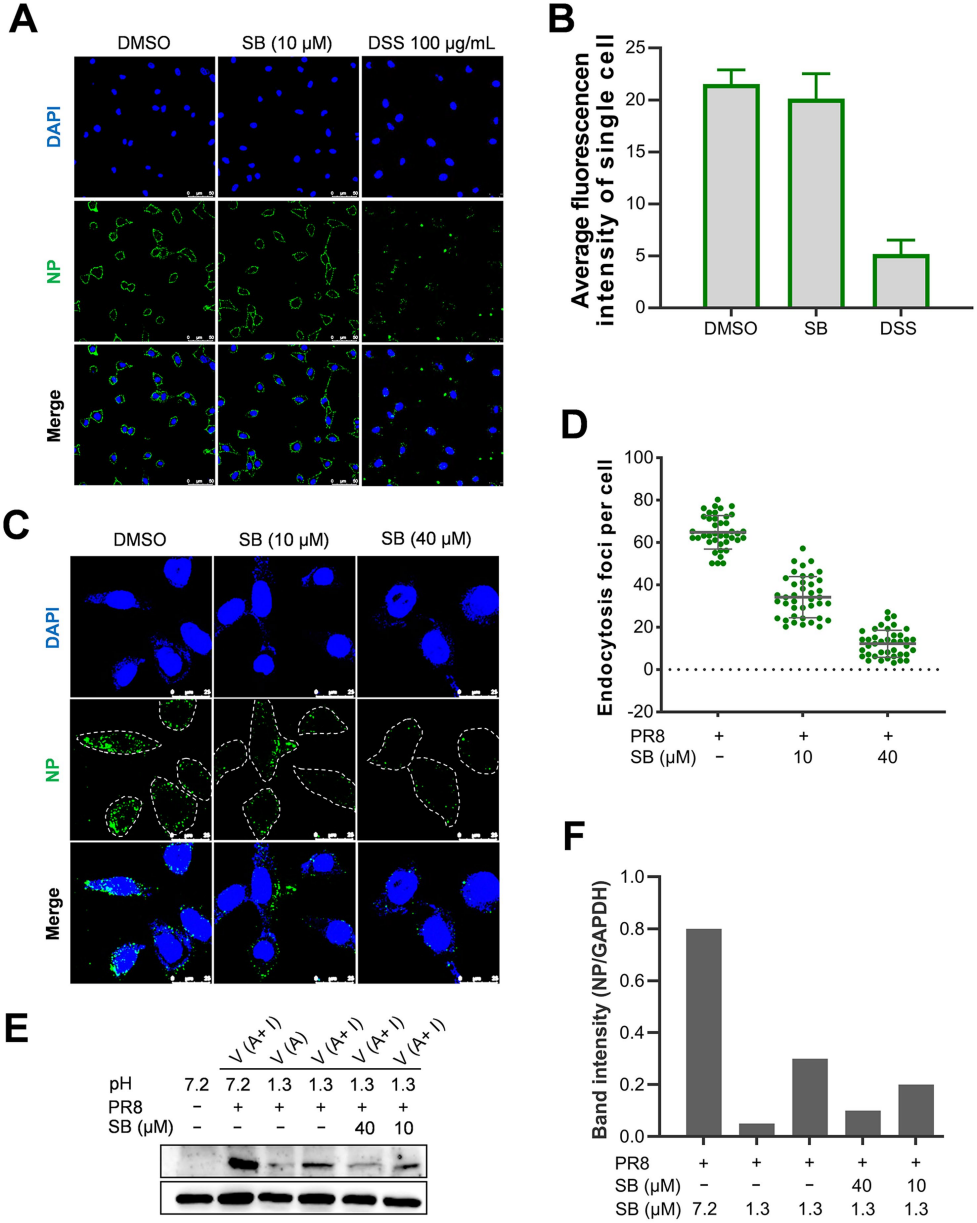
The impact of SB on the attachment and internalization of the influenza virus. **(A,B)** The effects of SB on viral attachment are examined. A549 cells were pre-cooled at 4 °C for 30 min, followed by infection with PR8 at 10 MOI in the presence or absence of 10 μM SB for 1 h at 4 °C. Subsequently, the cells were fixed using 4% PFA and stained utilizing a mouse anti-NP polyclonal antibody, followed by incubation with an Alexa Fluor 488-conjugated goat anti-mouse secondary antibody (green). Nuclei were counterstained with DAPI (blue). Scale bar: 50 μm. A representative IFA image is shown in panel **(A)**. The average green fluorescence intensities of individual cells in each group were obtained by analyzing green fluorescence from 100 cells using ImageJ software, which is represented as relative virus attachment in panel **(B)**. **(C–F)** The effects of SB on viral internalization were assessed by IFA and Western blotting. A549 cells were pre-cooled at 4 °C for 30 min before being infected with PR8 at 25 MOI in the presence or absence of SB at specified concentrations for 1 h at 4 °C. Subsequently, a temperature shift to 37 °C for 30 min was performed to facilitate internalization. The cells underwent two washes with cold phosphate-buffered saline (PBS; pH 7.2), followed by three washes with cold PBS-HCl (pH 1.3). NP was fluorescently stained (green) via IFA, and the nuclei were stained with DAPI (blue). A representative IFA image is shown in panel **(C)**. The quantification of endocytosis was determined by counting the number of NP fluorescent foci present in the cytoplasm per cell, as depicted in panel **(D)**. Values are expressed as means ± standard error of the mean (SEM) from three independent experiments. Viral internalization was evaluated using Western blot analysis **(E,F)**. Following viral attachment at 4 °C for one hour in the absence of 10 μM SB, the cells underwent two washes with cold PBS (pH 7.2). One group received three additional washes with cold PBS-HCl (pH 1.3) to remove unattached virions. Afterward, cell lysis and Western blotting were performed as a control to assess the removal efficiency of the attached virions. Other cell groups were transferred to 37 °C for 30 min to allow for internalization in the presence of 10 μM SB. Subsequently, the cells were lysed, and the levels of NP protein and GAPDH were assessed through Western blotting, as detailed in panel **(E)**. The quantitative analysis of the NP-to-GAPDH intensity ratio is shown in panel **(F)**. The results reported in **B** and **D** are representative of three independent experiments (mean ± SD). Statistically significant differences are indicated as ^*^p < 0.05, ^**^p < 0.01, and ^***^p < 0.001.

To determine whether SB interferes with the initial stage of viral replication, we evaluated the completion of each step in the early phase of viral replication after drug treatment. Initially, a virus attachment assay using IFA was performed to assess the localization and quantification of the viral nucleoprotein (NP). As illustrated in Figure 4A, treatment with SB did not impact the attachment of the influenza virus to A549 cells. Conversely, DSS, which has been documented to inhibit influenza viral attachment (Yamada et al., 2012), significantly obstructed viral attachment. The quantitative analysis of viral attachment presented in Figure 4B further corroborates that SB does not impede viral attachment, whereas the reference compound DSS does.

Subsequently, we employed IFA and Western blotting to evaluate the effects of SB on the internalization of influenza virions into A549 cells. As illustrated in Figure 4C, in the internalization assay using IFA to examine viral NP, virions that did not enter host cells were effectively removed by acidified PBS as described in the methods. In contrast, the internalized virions were visualized as distinct green dots within the cytoplasm or nuclei of the cells. Compared with untreated cells, which contained numerous internalized virions, treatment with SB effectively inhibited influenza virus internalization in a concentration-dependent manner during the infection. The quantitative analysis of the results in Figure 4C, presented in Figure 4D, clearly revealed a significant and concentration-dependent decrease in the number of internalized virions following SB treatment, as compared to the untreated control group.

To further substantiate the claim that SB inhibits the internalization of the influenza virus, we evaluated virion internalization using acid treatment, which effectively eliminates any virions that are not internalized. This was followed by Western blot analysis to quantify viral nucleoprotein (NP), a marker that correlates with the quantity of internalized virions. The results are illustrated in Figure 4E, which presents the Western blot analysis following acid treatment, and Figure 4F, which provides a quantitative analysis of the results in Figure 4E. The data indicate that SB treatment significantly reduces the levels of viral NP associated with internalized virions in the treated cells when compared to the untreated group.

Collectively, our results demonstrate that SB does not interfere with the attachment of influenza virus to A549 cells. Instead, SB exerts a potent inhibitory effect on the internalization of the virus into host cells during infection.

### 2.5 5-HT7R is required for the replication of influenza viruses

**Figure 5:**
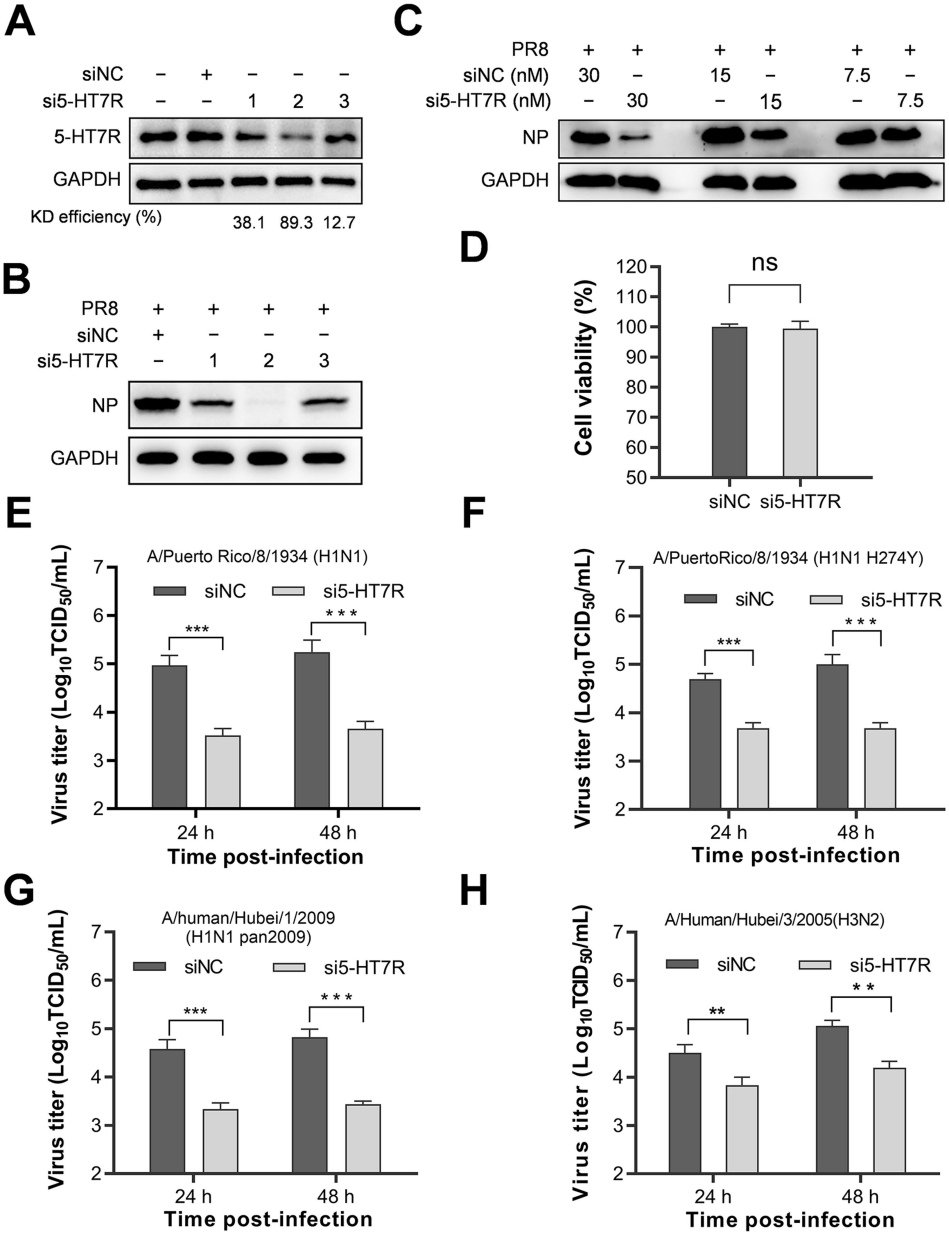
The involvement of 5-HT7R receptor in the replication of IAV within A549 cells. **(A)** A549 cells, cultured in 24-well plates, were subjected to transfection with 30 nM siRNA specifically targeting the 5-HT7R receptor (si5-HT7R) or a negative control siRNA (siNC) at 37 °C for 24 h. The knockdown efficiency was assessed by Western blot analysis of 5-HT7R and GAPDH proteins. **(B)** Following transfection with either si5-HT7R or siNC, A549 cells were infected with the PR8 strain at an MOI of 0.1 and subsequently cultured at 37 °C for an additional 24 h. The expression of NP was evaluated through Western blotting. **(C)** A549 cells were transfected with various concentrations of si5-HT7R or siNC under the same conditions prior to infection with PR8 at an MOI of 0.1 at 37 °C for 24 h. Following the infection, NP expression was determined via Western blotting. **(D)** In a separate experiment, A549 cells were transfected with 30 nM si5-HT7R or siNC at 37 °C for 48 h. Cell viability was assessed in each well utilizing the CellTiter-Glo kit. **(E–H)** A549 cells were transfected with 30 nM si5-HT7R or siNC at 37 °C for 24 h, followed by infection with PR8, PR8 (274Y), H1N1 (pan2009), and H3N2 strains, respectively, at an MOI of 0.1. At 24 and 48 hpi, cells and supernatants were collected, and virus titers were quantified using the TCID_50_ assay on MDCK cells. The results reported are representative of three independent experiments (mean ± SD). Statistically significant differences are represented as ^*^p < 0.05, ^**^p < 0.01, and ^***^p < 0.001. KD, knockdown.

To investigate the role of 5-HT7R in influenza virus replication, we depleted 5-HT7R expression using small interfering RNAs (siRNAs) in A549 cells. Subsequently, we assessed the effects of 5-HT7R knockdown on viral replication. The results, illustrated in Figure 5, demonstrate that the three siRNAs effectively diminished the expression of 5-HT7R (Figure 5A) and adversely impacted viral replication, as indicated by reduced levels of viral NP (Figure 5B). Notably, a correlation was identified between 5-HT7R expression levels and viral NP levels, suggesting that 5-HT7R plays a critical role in the replication of influenza viruses (Figures 5A,B).

To determine whether the cytotoxicity of siRNAs could influence the antiviral effect associated with knockdown of the 5-HT7R gene, we assessed the concentration-dependent effects of siRNA2. As shown in Figure 5C, the administration of siRNA 2 resulted in a dose-dependent reduction in viral NP expression. Furthermore, the administration of 30 nM siRNA2 did not elicit cytotoxiity in A549 cells, as demonstrated in Figure 5D. These findings confirm that 5-HT7R is a critical component in the replication process of the influenza virus PR8 strain.

Influenza viruses exhibit a significant capacity for mutation and recombination, leading to the emergence of diverse subtypes and strains in both human and animal populations. Our evaluation of the effects of 5-HT7R knockdown on the replication of several viral strains revealed a marked reduction in the production of infectious virions for both H1N1 and H3N2 strains, including a strain of H1N1 that is resistant to oseltamivir, as depicted in Figures 5E–H. These results provide strong evidence that 5-HT7R is integral to the replication process of influenza viruses.

### 2.6 The knockdown of 5-HT7R impairs the internalization of influenza viruses

**Figure 6:**
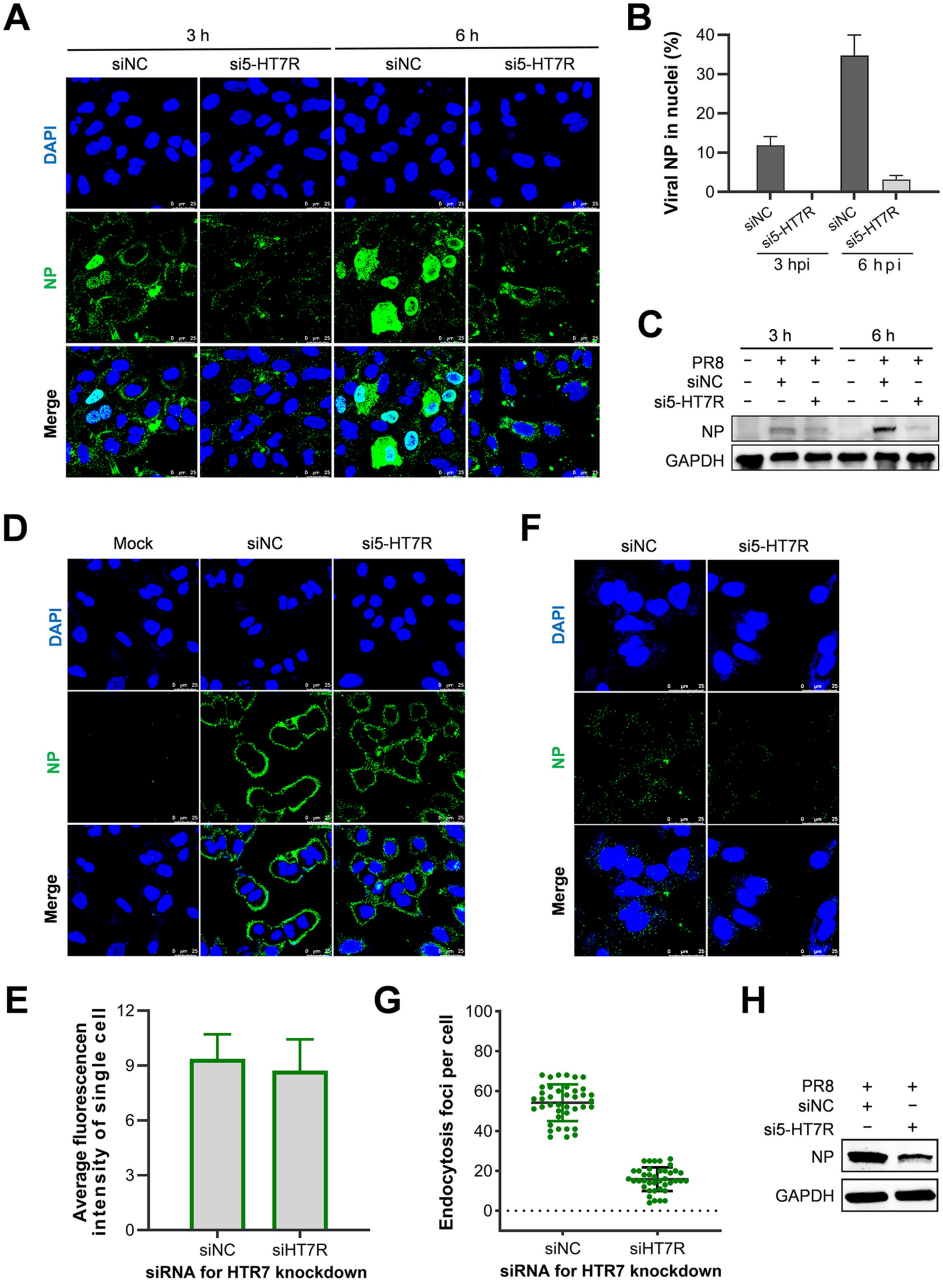
The impact of the 5-HT7R receptor on the internalization of influenza viruses. **(A–C)** The effects of 5-HT7R knockdown on the nuclear import of vRNP. A549 cells were transfected with either si5-HT7R or siNC at 37 °C for 24 h, followed by infection with the PR8 strain at 5 MOI. Samples were collected at 3 and 6 hpi and analyzed by IFA via confocal microscopy **(A)** and Western blot **(C)**. The scale bars represent 25 μm. The ratio of virus-infected cells exhibiting nuclear localization of viral NP in **A** was calculated based on the observation of about 100 cells **(B)**. **(D,E)** Influence of 5-HT7R knockdown on viral attachment. A549 cells were transfected with si5-HT7R or siNC at 37 °C for 24 h, followed by infection with PR8 at 25 MOI at 4 °C for 1 h. NP was fluorescently stained (green), while the nuclei were counterstained with DAPI (blue). The scale bar represents 25 μm. Viral attachment was evaluated by IFA targeting NP, and the average green fluorescence intensity per cell was quantified from 100 cells, as depicted in panel **(D)**, and was quantified using ImageJ software **(E)**. **(F–H)** Effects of 5-HT7R knockdown on endocytosis of influenza virions. A549 cells were transfected with either si5-HT7R or siNC at 37 °C for 24 h, followed by infection with PR8 at 25 MOI at 4 °C for 1 h. Subsequently, a temperature shift to 37 °C was employed for 30 min to facilitate virus internalization. The cells underwent two washes with cold PBS at pH 7.2, followed by three washes with cold PBS-HCl at pH 1.3. Samples were collected and subjected to IFA using confocal microscopy **(F)** and Western blot analysis **(H)**. The scale bar represents 25 μm. Endocytosis, as shown in panel **(F)**, was quantified by counting the number of NP fluorescent foci present in the cytoplasm per cell. Results are expressed as means ± standard error of the mean (SEM) derived from three independent experiments **(G)**. Viral NP levels associated with internalized virions in experiments conducted in **F** were analyzed using Western blotting **(H)**.

To examine the role of 5-HT7R in influenza virus replication, we assessed the impact of 5-HT7R knockdown on critical stages of the viral replication cycle. Initially, at 3 and 6 hpi, we evaluated the levels and localizations of viral NP using IFA. Our findings demonstrated that the knockdown of 5-HT7R substantially reduced NP expression (refer to Figure. 6A) and inhibited the nuclear import of viral vRNPs (see Figures 6A,B) at both time points. Furthermore, the expression levels of viral NP were significantly decreased in cells subjected to 5-HT7R knockdown when compared to those treated with siNC (as illustrated in Figure 6C). These findings suggest that knocking down 5-HT7R hinders influenza viral replication by blocking the processes involved in or before the nuclear import of vRNPs.

To enhance our understanding of the role of 5-HT7R in viral entry, we examined the effects of 5-HT7R knockdown on viral attachment and endocytosis in A549 cells infected with the influenza PR8 strain. As shown in Figure 6D, viral attachment was assessed by IFA targeting the viral NP. The findings indicated that the attachment of influenza viruses to A549 cells remained unaffected by the knockdown of 5-HT7R, a conclusion further supported by quantitative analysis of bound virions shown in Figure 6E.

Moreover, we evaluated the internalization of influenza viruses into A549 cells through IFA of viral NP. The results, depicted in Figure 6F, demonstrated that 5-HT7R knockdown significantly inhibited the endocytosis of virions into the cells. This observation was corroborated by quantitative analysis in Figure 6G, as well as Western blot analysis of the viral NP associated with endocytosed virions within cells, as shown in Figure 6H.

Collectively, these findings indicate that knocking down 5-HT7R does not affect influenza virus attachment. In contrast, 5-HT7R plays a significant role in facilitating viral internalization into host cells during viral infection. Notably, these results suggest that the compound SB inhibits the internalization of influenza viruses through a specific on-target mechanism.

### 2.7 SB inhibits the replication of the influenza virus by inhibiting PLC activation

**Figure 7:**
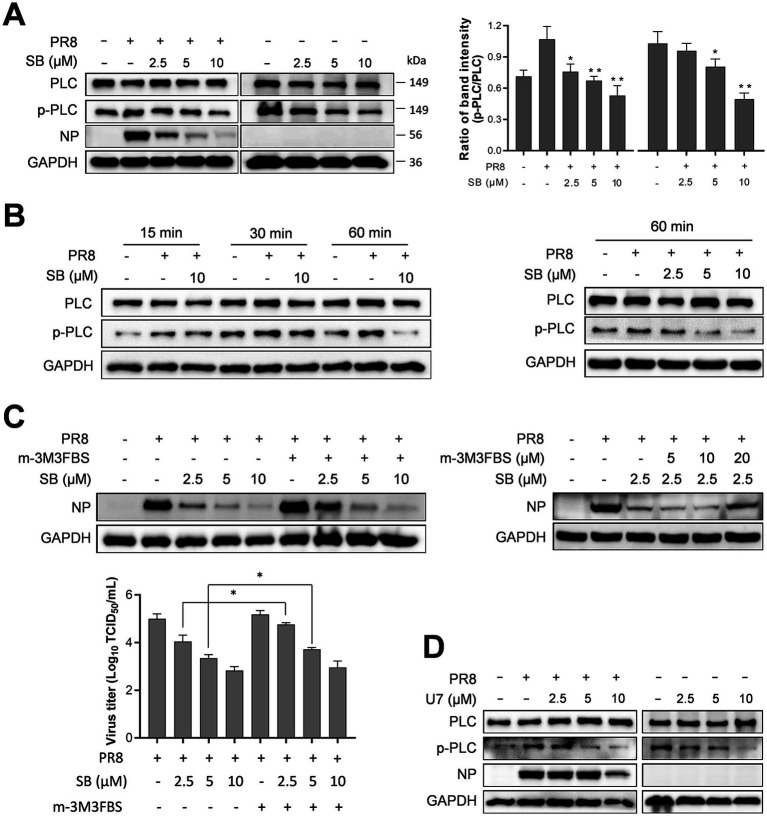
The inhibitory effects of SB on PR8 virus replication through PLC inhibition. **(A)** Suppression of PLC activation and antiviral efficacy of SB against influenza viral replication. A549 cells were infected with PR8 at an MOI of 0.1 in the presence or absence of various concentrations of SB at 37 °C for 1 h. Subsequently, the cells were subjected to three washes with fresh medium; the final wash contained varying concentrations of SB. Another set of A549 cells was treated with various concentrations of SB without viral infection (left panel). After incubation at 37 °C for 24 h, samples were collected, and the phosphorylation of PLC, along with viral NP, was analyzed via Western blotting. A quantitative analysis of the p-PLC/PLC ratio is shown in the right panel. **(B)** The impact of influenza viral infection and SB treatment on PLC activation. A549 cells were exposed to PR8 at an MOI of 10 in the presence or absence of 10 μM SB at 37 °C for 15, 30, or 60 min (left panel). Another set of A549 cells was infected with PR8 at an MOI in the presence or absence of varying concentrations of SB at 37 °C for 60 min (right panel). The indicated proteins were subsequently detected by Western blotting. **(C)** Restoration of viral replication suppressed by SB treatment through *m*-3M3FBS treatment. A549 cells were treated with *m*-3M3FBS at 37 °C for 1 h, followed by two washes with phosphate-buffered saline (PBS). These cells were then infected with PR8 at an MOI of 0.1 in the presence or absence of SB at 37 °C for 1 h. Following this, the cells were treated with fresh medium containing various concentrations of SB. After a 24-h incubation at 37 °C, samples were collected, and the relevant proteins were analyzed by Western blotting (left panel). A separate group of A549 cells was treated with 2.5 μM of SB, along with varying concentrations of *m*-3M3FBS (right panel), and viral NP levels were subsequently assessed by Western blotting (left lower panel). **(D)** Inhibition of PLC activation and viral replication by U7. A549 cells were either infected (left panel) or uninfected (right panel) with PR at an MOI of 0.1 and treated with various concentrations of U7 at 37 °C for 1 h. Following treatment, cells were washed and incubated with fresh medium containing the indicated concentrations of U7. The samples were then collected, and the relevant proteins were examined via Western blotting.

Host factor phospholipase C (PLC) is known for its role in facilitating the cellular entry of the H1N1 influenza strain (Zhu et al., 2014). To explore whether SB inhibits PR8 infection by modulating PLC activation, we first examined its impact on PLC phosphorylation in PR8-infected and uninfected A549 cells. As shown in Figure 7A (left), PR8 infection led to PLC phosphorylation, indicating activation of the PLC pathway, which was suppressed by SB treatment. A similar inhibitory effect on PLC phosphorylation was observed in uninfected A549 cells (Figure 7A, right).

To determine whether the suppression of PLC by SB is linked to the inhibition of viral replication, we examined PLC phosphorylation at early stages post-infection (within 1 h), before new virion production began. As illustrated in Figure 7B, PLC phosphorylation was detectable as early as 15 min after viral infection, with further increases observed at 30 min and 1 h. Treatment with 10 μM SB significantly reduced virus-induced PLC activation. These findings indicate that PLC is activated in the early stages of IAV infection and that SB inhibits IAV-induced PLC activation in MDCK cells.

As shown in the left panel of Figure 7C, SB displayed significant anti-IAV activity in the untreated cells. However, pretreatment with *m*-3M3FBS, a direct activator of PLC, for 1 h partially reversed the antiviral effects at lower SB concentrations (2.5 and 5 μM). Additional experiments revealed that *m*-3M3FBS could dose-dependently restore viral replication, as indicated by NP levels and viral titers (Figure 7C). These findings suggest that antiviral activity is linked to the suppression of PLC activation.

To rule out any off-target effects that might contribute to the antiviral activity of SB, we evaluated the antiviral efficacy of U73122 (U7), a PLC inhibitor, and its effect on PLC phosphorylation in both PR8-infected and uninfected A549 cells. Infected cells treated with U7 showed a significant suppression of viral NP expression and inhibition of virus-induced PLC activation (Figure 7D, left). Similarly, U7 decreased PLC phosphorylation in uninfected A549 cells (Figure 7D, right).

In summary, our findings reveal that anti-IAV activity is facilitated by inhibition of PLC activation. The antiviral effect of SB was diminished by exogenous PLC activation using *m*-3M3FBS, resulting in restored viral replication. This finding further underscores the functional link between SB and the PLC pathway in the context of IAV infection.

## 3 Discussion

Influenza viruses pose a significant and ongoing global health threat owing to their high mutation rates, antigenic drift and shift processes, and frequent emergence of drug-resistant variants. These factors compromise the effectiveness of current antiviral treatments, such as neuraminidase inhibitors (e.g., oseltamivir) and M2 ion channel blockers (e.g., adamantanes) (Bright et al., 2005; Deyde et al., 2007; Hayden et al., 2018). These challenges highlight the urgent need for innovative therapeutic interventions targeting host factors essential for viral replication. Such an approach could reduce the potential for resistance development and offer broader antiviral coverage (Karlas et al., 2010; Ji and Li, 2020). In this study, we provide evidence that SB, a selective antagonist of the serotonin 5-HT7 receptor, effectively inhibits the replication of various influenza A and B virus strains, including an oseltamivir-resistant H1N1 variant, by specifically blocking the viral internalization. Our findings identify 5-HT7R as a previously unrecognized host dependency factor for influenza virus entry and position SB as a promising candidate for host-directed antiviral therapy.

Our *in vitro* studies revealed that SB effectively inhibited the replication of H1N1 (including both the PR8 and pandemic 2009 strains), influenza B virus, and an oseltamivir-resistant H1N1 strain in human lung epithelial (A549) and canine kidney (MDCK) cells. The effective concentrations (EC_50s_) are observed in the low micromolar range, with selectivity indices (SI) exceeding 50. However, the H3N2 strain showed significantly reduced sensitivity to SB, with an EC_50_ greater than 56 μM in MDCK cells and an SI of approximately 5.7. This finding suggests potential subtype-specific differences in the mechanisms of viral entry or connectivity to the 5-HT7R. This observation aligns with previous investigations, indicating that distinct influenza subtypes may employ varied endocytic pathways or rely on specific host cofactors for their internalization (Sieczkarski and Whittaker, 2002; Zhu et al., 2014). For instance, while H1N1 predominantly utilizes clathrin-mediated endocytosis, certain H3N2 strains may employ alternative or redundant mechanisms, which could account for their diminished susceptibility to SB inhibition.

Time-of-addition and early infection assays have pinpointed the antiviral effect of SB to the initial 0–2 h post-infection. This critical period aligns with the processes of viral attachment and internalization in the host cells. Importantly, SB did not interfere with viral binding to host cells, as demonstrated by immunofluorescence at 4 °C. However, it significantly inhibited post-attachment internalization, as demonstrated by acid-wash/Western blot and confocal microscopy. This specific phenotype was replicated by siRNA-mediated knockdown of 5-HT7R, which similarly blocked viral endocytosis while leaving attachment unaffected. These complementary genetic and pharmacological findings strongly support an on-target mechanism in which 5-HT7R facilitates influenza virus internalization, likely by modulating downstream signaling pathways that regulate the endocytic machinery.

Our data mechanistically implicate PLC as a key signaling node downstream of 5-HT7R during influenza virus entry. Treatment with SB effectively suppressed both the basal levels and virus-induced phosphorylation of PLC, showing a temporal correlation with the blockade of viral internalization. Notably, pharmacological activation of PLC via *m*-3M3FBS partially rescued viral replication in SB-treated cells, whereas direct inhibition of PLC using U7 mimicked the antiviral effects observed with SB treatment. These findings establish a functional relationship between 5-HT7R signaling, PLC activation, and endocytosis of the influenza virus. Given that 5-HT7R is a Gαs-coupled G protein-coupled receptor (GPCR) known to modulate cAMP/PKA and MAPK pathways (Thomas and Hagan, 2004; Lieb et al., 2005), it is plausible that interactions between these pathways and PLCγ1, previously implicated in H1N1 viral entry (Zhu et al., 2014), are responsible for the observed phenotype. Future research should aim to elucidate the specific signaling intermediates that connect 5-HT7R to clathrin-coated pit formation or actin remodeling during viral uptake.

The *in vivo* efficacy of SB further highlights its therapeutic potential. In a lethal murine influenza model, both prophylactic and early therapeutic administration of SB significantly improved survival, with increases of up to 70% at a dosage of 7.5 mg/kg/day. Additionally, SB reduced lung viral titers and alleviated histopathological lung damage. The observed dose-dependent protection, with optimal efficacy at 7.5 mg/kg/day and a slight decrease in survival at 10 mg/kg/day, suggests a narrow therapeutic window that requires further optimization of the pharmacokinetics and toxicology. Notably, the ability of SB to reduce viral load and inflammation in pulmonary tissues aligns with its mechanism of action, which targets the initial stages of infection. This approach effectively limits both direct cytopathic effects and subsequent immunopathological responses, a dual advantage not commonly achieved by late-acting antiviral agents (Ludwig, 2011).

Additionally, the antiviral concentration range of SB269970 *in vitro* (1–5 μM) aligns with plasma concentrations achieved in mice at standard *in vivo* doses of 10–30 mg/kg (Hagan et al., 2000; Hedlund et al., 2005). While direct pharmacokinetic data for mouse lung tissue are limited, lung exposures are likely to reach effective levels based on the compound’s known pharmacokinetic profile. Studies confirm that SB269970 crosses the blood–brain barrier, with a reported brain-to-plasma concentration ratio of approximately 0.5–0.8 (Forbes et al., 2002).

Targeting host factors, such as the 5-HT7 receptor (5-HT7R), offers several strategic advantages over traditional antiviral therapies. First, host-directed inhibitors are less prone to driving rapid resistance, as they do not exert direct selective pressure on the viral genome (Ji and Li, 2020). Second, these agents may also confer broad-spectrum efficacy against diverse influenza strains, including those resistant to current antiviral agents, as observed with the oseltamivir-resistant H1N1 variant. Additionally, repurposing well-characterized compounds, such as SB, can accelerate preclinical development.

However, targeting 5-HT7R presents notable challenges due to its widespread distribution in central and peripheral tissues, where it regulates key physiological processes including circadian rhythm, vascular tone, and gastrointestinal function (Hagan et al., 2000; Roberts and Hedlund, 2012). Systemic antagonism therefore carries a risk of off-target effects. While the prototypical antagonist SB269970 is well-tolerated in acute studies, its central nervous system (CNS) penetration raises safety concerns for systemic administration in non-CNS indications (Monti and Jantos, 2014). Documented effects include altered sleep architecture and subtle behavioral changes (Roberts and Hedlund, 2012; Monti and Jantos, 2014). These CNS-related effects are consistent with prior pharmacological studies of 5-HT7 receptor modulation, which have demonstrated its involvement in sleep-wake cycles, memory consolidation, and other behavioral parameters (Gasbarri et al., 2008; Hedlund and Sutcliffe, 2004; Monti and Jantos, 2008). Consequently, development strategies that prioritize tissue-selective delivery, such as inhaled formulations to maximize lung bioavailability while minimizing systemic and CNS exposure, are crucial for mitigating these risks while preserving antiviral efficacy.

In conclusion, our study identified the serotonin 5-HT7 receptor as a novel host factor crucial for the internalization of the influenza virus. Furthermore, our findings demonstrate that pharmacological blockade of this receptor using SB exhibits significant antiviral efficacy both *in vitro* and *in vivo*. By targeting the entry stage through a phospholipase C-dependent mechanism, SB offers a promising host-targeted therapeutic strategy against influenza, particularly given growing concerns regarding antiviral resistance. Future research should aim to clarify the structural and signaling interactions between the 5-HT7 receptor and the influenza virus. We will also evaluate the effectiveness of SB in combination with existing antiviral agents, such as baloxavir and oseltamivir, and explore its efficacy against emerging influenza variants and other respiratory viruses that utilize similar entry mechanisms.

## 4 Materials and methods

### 4.1 Cell lines and virus strains

The Human Pulmonary Epithelial (A549) cells (ATCC CCL-185) and the Madin–Darby Canine Kidney (MDCK) cells (ATCC CCL-34) were cultured in Dulbecco’s Modified Eagle’s Medium (DMEM, Gibco, Grand Island, NY, United States). The DMEM was supplemented with 10% fetal bovine serum (FBS, Meilunbio, Dalian, China), along with penicillin and streptomycin at concentrations of 100 U/mL each (Gibco, Rockville, MD, United States). All cell cultures were maintained at 37 °C in an incubator with 5% CO_2_.

The influenza virus strains utilized included A/Puerto Rico/8/1934 (H1N1), an Oseltamivir-resistant variant of A/Puerto Rico/8/1934 (H1N1 OST-R), A/human/Hubei/1/2009 (H1N1 pan2009), A/human/Hubei/3/2005 (H3N2), and B/human/Hubei/1/2007 (IBV). These strains were provided by the National Virus Resource Center in Wuhan, China.

### 4.2 Compounds

The compounds SB HCl, U-73122, and *m*-3M3FBS were obtained from TopSience (Shanghai, China). Stock solutions were prepared using dimethyl sulfoxide (DMSO) and stored at −40 °C. For each compound, working concentrations were generated by diluting the stock solutions in either culture medium or reaction buffer immediately before use.

### 4.3 Antibodies

Influenza A nucleoprotein antibody (NP) (Sino Biological, Beijing, China), 5-HT7R receptor antibody (Affinity Biosciences, Jiangsu, China), PLCγ1 Rabbit mAb, and Phospho-PLCγ1 (Ser1248) Rabbit mAb were acquired from Selleck (Beijing, China). The secondary antibody, goat anti-mouse conjugated to Alexa Fluor^®^ 488 (green), was purchased from Cell Signaling Technology, Inc. (Massachusetts, United States). Additionally, the GAPDH mouse monoclonal antibody, as well as the HRP-labeled goat anti-mouse IgG (H + L) and HRP-labeled goat anti-rabbit IgG (H + L) secondary antibodies, were sourced from Beyotime Biotechnology (Shanghai, China). Furthermore, the DAPI solution was obtained from Solarbio (Beijing, China).

### 4.4 Cytotoxicity assays

A549 or MDCK cells were cultured in 96-well plates at a density of 25,000 cells per well. After a 24-h incubation period, the cells were exposed to the test compounds at specified concentrations for 48 h. Cell viability was assessed using the Cell Titer-Glo luminescent cell viability assay (Promega, Madison, WI, United States). Luminescent signals were recorded using a Varioskan LUX multimode microplate reader (Thermo, Waltham, MA, United States), and cell viability was normalized to the vehicle-treated control. The CC_50_ values were determined utilizing GraphPad Prism 10.0.

### 4.5 Antiviral assay

A549 or MDCK cells were cultured in 96-well plates at a density of 20,000 cells per well. After a 24-h incubation period, the cells were infected with PR8 at a multiplicity of infection (MOI) of 0.1 or 0.01, in the presence of varying concentrations of SB. The cells were subsequently incubated at 37 °C for 1 h, followed by treatment with fresh medium containing the specified concentrations of SB for an additional 24 h. The inhibition of viral replication was evaluated using a modified neuraminidase (NA) activity assay, as described by Ivachtchenko et al. (2013). Luminescence signals were detected using a Varioskan LUX multimode microplate reader (Thermo, Waltham, MA, United States).

### 4.6 Measurement of virus titer

Viral titers were determined using the TCID_50_ assay. MDCK cells were cultured in 96-well plates at a density of 20,000 cells per well. Following a 24-h incubation period, tenfold serial dilutions of the virus-containing supernatants were inoculated onto the MDCK monolayer at 37 °C for 2 h. Subsequently, the medium was replaced with fresh Dulbecco’s Modified Eagle Medium (DMEM), and the cells were cultured for an additional 72 h. The endpoint was assessed based on neuraminidase (NA) activity, and viral titers were calculated using the approach established by Reed and Muench (1938).

### 4.7 Indirect immunofluorescence assay (IFA)

A549 cells were subjected to a wash with phosphate-buffered saline (PBS) and subsequently fixed in 4% paraformaldehyde (PFA) for a duration of 30 min at room temperature. Following three washes with PBS, the cells were permeabilized using 0.25% Triton X-100 in PBS for 15 min at room temperature. The permeabilized cells were then blocked with 5% bovine serum albumin (BSA) in PBS for 1 h at 37 °C, after which they were incubated overnight at 4 °C with mouse anti-NP antibodies. Following three additional washes with PBS, the cells were treated with Alexa Fluor 488-conjugated goat anti-mouse immunoglobulin G (IgG) (H + L) for 1 h. Upon completion of three washes, the cells were incubated with DAPI (4′,6-diamidino-2-phenylindole) for 5 min to stain the nuclei. After three final washes with PBS, immunofluorescence was visualized utilizing either a Leica DMI 4000B fluorescence microscope or a TCS SP8 confocal laser scanning microscope (Leica, Wetzlar, Germany).

### 4.8 Virus attachment and internalization assays

In the viral attachment assay, A549 cells were incubated at 4 °C for 1 h prior to the addition of 1 mL of virus solution containing SB at 10 μM. After 1 h of adsorption at 4 °C, the cells were washed three times with PBS to remove unbound virus particles. Subsequently, the cells were analyzed for viral NP using a laser scanning confocal microscope.

For the viral internalization assay, as described by Wang et al. (2020), the inhibition of virus internalization was evaluated using the acid wash method, and the specific procedure was as follows: A549 cells were incubated at 4 °C for 1 h and subsequently infected with the PR8 virus, with or without SB at the specified concentration. After 1 h of adsorption at 4 °C, the cells were transferred to 37 °C for 30 min to facilitate internalization. Following this period, the cells were washed three times with ice-cold PBS at pH 1.3 to remove any attached but non-internalized virions. The cells were then lysed with SDS-PAGE loading buffer (Beyotime Biotechnology, Shanghai, China) and subjected to Western Blotting for the detection of NP, or fixed with 4% paraformaldehyde for the detection of NP by confocal microscopy. Viral internalization is assessed by manual counting of NP foci. Individual cell boundaries are meticulously delineated, followed by the manual enumeration of NP puncta. For each experimental assessment, a minimum of 50 cells is analyzed.

### 4.9 Western blotting assay

Cells were rinsed three times with ice-cold PBS and subsequently lysed in RIPA buffer (Beyotime Biotechnology, Shanghai, China). The resulting cell lysate was centrifuged at 4 °C for 10 min at 12,000 × g, and the supernatant was carefully collected. The supernatants were boiled for 10 min, and 10 μg of lysate was loaded onto an SDS gel. Membranes, blocked with 5% skim milk in Tris-buffered saline with Tween 20 (TBST), were incubated at room temperature for 1 h. This was followed by an overnight incubation at 4 °C with primary antibodies specific to the influenza NP at 1:1,000 and glyceraldehyde-3-phosphate dehydrogenase (GAPDH) at 1:1,000. After this incubation, a horseradish peroxidase (HRP)-conjugated secondary antibody, diluted to 1:2,000, was applied. Target proteins were subsequently visualized utilizing the Clarity Western ECL Substrate (Bio-Rad, Singapore), and the resulting signals were captured employing a ChemiDoc^™^ imaging system (Bio-Rad, Singapore).

### 4.10 siRNA transfection

Small interfering RNAs (siRNAs) targeting the 5-HT7 receptor were synthesized by Sangon Biotech. A549 cells were cultured in 24-well plates and subsequently transfected with 30 nM of si5-HT7 receptor (si5HTR) or a non-targeting siRNA (siNC), using 1 μL of Lipofectamine 3000 Transfection Reagent (Thermo Fisher Scientific, CA, United States). The siRNA sequences are as follows: si5-HT7R-1, 5′-GAAAGUUGUGAUCGGCUCCAU-3′; si5-HT7R-1 antisense, 5′-AUGGAGCCGAUCACAACUUUC-3′; si5-HT7R-2, 5′-GCUCAGAAUGUAAAUGAUGAU-3′; si5-HT7R-2 antisense, 5′-AUCAUCAUUUACAUUCUGAGC-3′; si5-HT7R-3, 5′-GAAAGUUGUGAUCGGCUCCAU-3′; si5-HT7R-3 antisense, 5’-UUUGUAGCACAAACUCAGGUC-3′; the non-targeting control siRNA (siNC), 5′-UUCUCCGAACGUGUCACGUTT-3′.

### 4.11 IAV animal experiment

Six to eight-week-old female BALB/c mice were procured from the Guangdong Medical Laboratory Animal Center (GDMLAC, Guangdong, China) and were housed in a specific pathogen-free environment. The compounds were resuspended in a solution of 0.9% sodium chloride containing 2.5% dimethyl sulfoxide (DMSO). Ten mice per group received SB at doses of 5, 7.5, or 10 mg/kg/day. The treatment was administered 30 min before intranasal infection with the PR8 strain (5 × LD_50_) after the mice were anesthetized using inhaled isoflurane (3% in oxygen). Subsequently, the mice received intraperitoneal injections of SB or a control solution containing 0.9% sodium chloride and 2.5% DMSO, administered twice daily for 30 days. Body weight and survival rates were monitored daily for 14 days post-infection. For lung tissue collection, the mice were deeply anesthetized using isoflurane inhalation (5% in oxygen) and euthanized by cervical dislocation. All collected lung tissues were fixed in 4% paraformaldehyde on day 5 post-infection. H&E-stained slides were examined for tissue damage, necrosis, and inflammatory cell infiltration. Sections of paraffin-embedded lung tissue were further stained with H&E and subsequently observed under a microscope at a magnification of 200×.

### 4.12 Preparation of lung tissue homogenates

Mice were humanely euthanized, and lung tissue was aseptically collected. The lung tissue was transferred to a centrifuge tube containing a suitable volume of phosphate-buffered saline (PBS), from typically 1 to 2 mL per 100 mg of tissue. A tissue grinder was used to homogenize the sample at 4 °C. Following homogenization, the specimen was centrifuged at 5,000 g for 15 min at 4 °C, and the supernatant was subsequently collected for further analysis.

### 4.13 Statistical analysis

Quantitative data are presented as means ± standard deviations (SDs) derived from three independent experiments. Statistical analyses were conducted using Student’s *t*-test for the comparison of two groups and one-way analysis of variance (ANOVA) for comparisons involving more than two groups. Statistical significance was determined with thresholds of ^*^p < 0.05, ^**^p < 0.01, and ^***^p < 0.001. These criteria were deemed statistically significant.

## Data Availability

The original contributions presented in the study are included in the article/supplementary material, further inquiries can be directed to the corresponding author.
